# Supplementary material for: In vitro fusion of single synaptic and dense core vesicles reproduces key physiological properties
Source: Nat Commun. 2019 Aug 29;10:3904. doi: 10.1038/s41467-019-11873-8 (PMC6715626; doi:10.1038/s41467-019-11873-8)
Supplement: Supplementary file 1 — Supplementary Information [file 41467_2019_11873_MOESM1_ESM.pdf]

Supplementary Information

Supplementary Table 1: Summary of experiments for SNARE-mediated docking and fusion of synaptic vesicles (SV), dense core vesicles (DCV), and insulin vesicles (INS). Related to data in Figure 3. Errors for normalized docking and percent fusion are standard errors.

**Condition**	**Secretory Vesicle**	**Number of Experiments**	**Normalized Docking**	**Percent Fusion**	**Number of Docking Events**	**Number of Fusion Events**
syx(183-288):dSN25	SV	5	1.0 ± 0.2	24 ± 4	224	53
	DCV	14	1.0 ± 0.1	41.0 ± 0.9	823	340
	INS	5	1.0 ± 0.2	32 ± 4	327	111
syx(1-288):dSN25	SV	5	0.5 ± 0.1	11 ± 1	114	13
	DCV	5	0.3 ± 0.1	30 ± 2	160	49
	INS	4	0.45 ± 0.10	20 ± 4	117	23
syx(183-288)	SV	5	0.13 ± 0.06	--	27	2
	DCV	5	0.14 ± 0.02	--	38	4
	INS	5	0.08 ± 0.02	--	27	1
syx(1-288)	SV	5	0.09 ± 0.04	--	19	1
	DCV	5	0.03 ± 0.02	--	17	1
	INS	5	0.07 ± 0.04	--	24	1
dSN25	SV	5	0.10 ± 0.04	--	21	3
	DCV	5	0.15 ± 0.04	--	27	1
	INS	5	0.11 ± 0.04	--	35	1
no protein	SV	5	0.09 ± 0.02	--	18	1
	DCV	5	0.19 ± 0.04	--	41	4
	INS	5	0.09 ± 0.06	--	30	1
syb(1-96) inhibitor	SV	5	0.02 ± 0.02	--	7	1
	DCV	5	0.10 ± 0.04	--	19	2
	INS	5	0.06 ± 0.03	--	19	1
syx(1-288):dSN25 0.5 μM Munc18 2 μM complexin-1	SV	5	0.8 ± 0.2	7 ± 3	244	17
	DCV	5	0.8 ± 0.1	2 ± 1	203	6
	INS	5	0.9 ± 0.1	4 ± 1	220	9

Supplementary Table 2: Summary of experiments for calcium stimulated fusion of different secretory vesicle types related to data shown in Figure 4 and 5A. All data was collected in the presence of syntaxin-1a (1-288):dSNAP-25, Munc18 and complexin-1. Data is shown in the absence and presence of C1C2MUN of Munc13. The delay time for the buffer exchange (Δt_{Ca}) with standard deviation, as illustrated in Supplementary Figure 3, is given for each condition. Errors for fusion percent and delay times are standard errors.

**Condition**	**Secretory Vesicle**	**Number of Experiments**	**Fusion (%)**	**Number of Docking Events**	**Number of Fusion Events**	**Δt_{Ca} (s)**
No C1C2MUN	SV	5	21 ± 3	316	69	0.14 ± 0.07
	DCV	5	63 ± 2	723	462	0.5 ± 0.4
	INS	5	49 ± 2	472	231	0.24 ± 0.09
	DCV CAPS KD	5	20 ± 3	701	147	0.3 ± 0.1
0.2 μM C1C2MUN	SV	5	77 ± 3	222	171	0.2 ± 0.1
	DCV	5	70 ± 3	233	161	0.24 ± 0.9
	INS	5	59 ± 3	215	125	0.3 ± 0.1
	DCV CAPS KD	5	70 ± 2	524	375	0.25 ± 0.05

Supplementary Table 3: Summary of insulin fusion kinetics from time of calcium arrival for three different labeling strategies. Related to data shown in Supplementary Figure 5. Errors for fusion percent and delay times are standard errors. Errors for the rate constant k (s^{-1}) are fit errors.

**Label**	**Number of Experiments**	**Fusion (%)**	**Number of Docking Events**	**Number of Fusion Events**	**k (s^{-1})**	**Δt_{Ca} (s)**
C-peptide-GFP	5	58.7 ± 2.6	215	125	0.11 ± 0.01	0.3 ± 0.1
NPY-mRuby	5	58.2 ± 1.5	124	72	0.11 ± 0.01	0.2 ± 0.08
Acridine Orange	5	58.6 ± 1.8	162	72	0.12 ± 0.01	0.2 ± 0.1

Supplementary Table 4: Summary of fusion kinetics of dense core vesicles expressing single synaptotagmin isoforms. Related to data shown in Figure 5B. Errors for fusion percent and delay times are standard errors. Errors for the rate constant k (s^{-1}) are fit errors.

Synaptotagmin Isoform	Number of Experiments	Fusion (%)	Number of Docking Events	Number of Fusion Events	k (s^{-1})	Δt_{Ca} (s)
Syt-1	5	63.7 ± 1.7	227	145	0.17 ± 0.02	0.2 ± 0.1
Syt-7	5	66.3 ± 3.1	189	126	0.34 ± 0.01	0.18 ± 0.09
Syt-9	5	69.0 ± 2.6	309	209	0.19 ± 0.01	0.15 ± 0.06

Supplementary Table 5: Summary of difference secretory vesicles docking in the presence of plasma membrane SNARE inhibitor complex (syx(191-253) and SNAP-25). Related to data shown in Figure 5C. These docking experiments were performed in the presence of Munc18 and complexin-1, in the absence of calcium, i.e. conditions that result in minimal numbers of calcium independent fusion events (1). Errors for normalized docking are standard errors.

**syx(191-253): SNAP-25**	**Secretory Vesicle**	**Number of Bilayers**	**Normalized Docking**	**Number of Docking Events**	**Number of Fusion Events**
0	SV	4	1.0 ± 0.2	181	2
	DCV	5	1.00 ± 0.05	324	8
	INS	4	1.0 ± 0.2	192	5
0.25	SV	5	0.51 ± 0.06	115	1
	DCV	3	0.69 ± 0.04	134	1
	INS	5	0.80 ± 0.03	192	1
0.5	SV	5	0.26 ± 0.01	58	0
	DCV	5	0.41 ± 0.1	134	2
	INS	5	0.53 ± 0.1	126	2
0.75	SV	5	0.10 ± 0.02	23	0
	DCV	5	0.16 ± 0.06	52	0
	INS	5	0.25 ± 0.08	49	1
1	SV	4	0.04 ± 0.02	8	0
	DCV	5	0.06 ± 0.02	18	0
	INS	5	0.15 ± 0.06	37	0
2	SV	3	0.03 ± 0.03	3	0
	DCV	3	0.01 ± 0.02	2	0
	INS	4	0.04 ± 0.02	5	0

Supplementary Table 6: Summary of fusion of secretory vesicles docked in the presence of 0.5 μM syx(191-253):SNAP-25 triggered with 100 μM calcium. Related to data shown in Figure 5D. Errors for fusion percent and delay times are standard errors. Errors for the rate constant k (s^{-1}) are fit errors.

**Secretory Vesicle**	**Number of Experiments**	**Fusion (%)**	**Number of Docked Vesicles**	**Number of Fusion Events**	**k (s^{-1})**	**Δt_{Ca} (s)**
SV	8	77.0 ± 1.4	208	160	3.7 ± 0.21	0.17 ± 0.06
DCV	8	67.7 ± 2.3	189	130	0.31 ± 0.01	0.2 ± 0.1
INS	8	60.6 ± 2.0	292	180	0.11 ± 0.01	0.2 ± 0.2

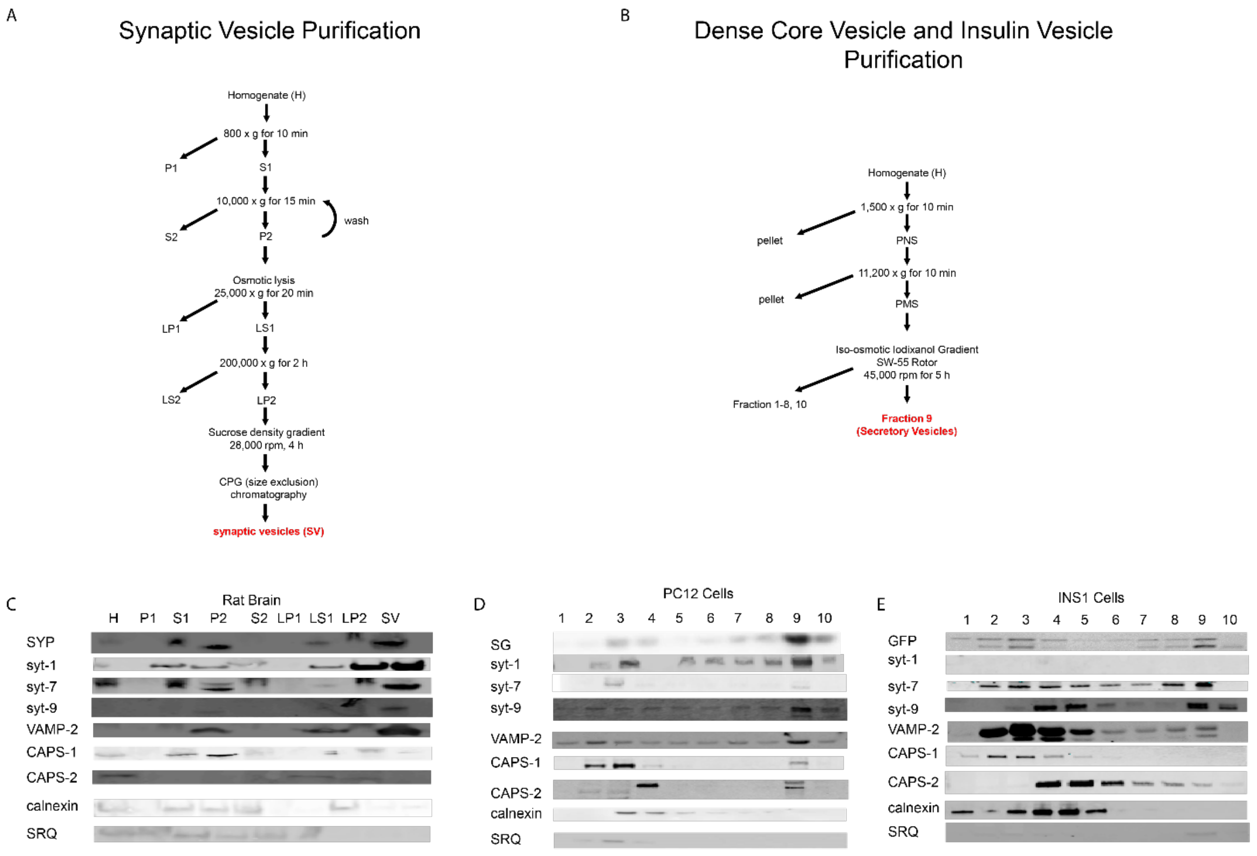

Supplementary Figure 1: Purification of secretory vesicles. (A) Schematic of purification scheme used for synaptic vesicles (adapted from ref. (2)) or (B) PC12 dense core vesicles and INS1 insulin vesicles (adapted from ref. (1)). Western Blots were run to determine purity and protein content of (C) synaptic vesicle purification from rat brain, (D) PC12 cell dense core vesicle iodixanol gradients, and (E) INS1 cell insulin vesicles iodixanol gradients. The vesicle markers synaptophysin (SYP) and secretogranin II (SG) were used as markers for synaptic vesicles and PC12 cell dense core vesicles, respectively. GFP was used to mark the C-peptide-GFP in the insulin vesicles with proinsulin as the top band and C-peptide as the lower band. (C) The lane labeled SV was the purified synaptic vesicles, while the 9th fraction sitting on top of a 0.5 mL 30% iodixanol layer was the sample of purified PC12 dense core vesicles (D) or INS1 insulin vesicles (E). Synaptotagmin isoforms -1, -7, and -9 (syt-1, syt-7, and syt-9), VAMP-2, CAPS-1, CAPS-2, calnexin (endoplasmic reticulum marker), and succinate ubiquinone oxidoreductase (SRQ, a mitochondrial marker) were blotted in all purifications.

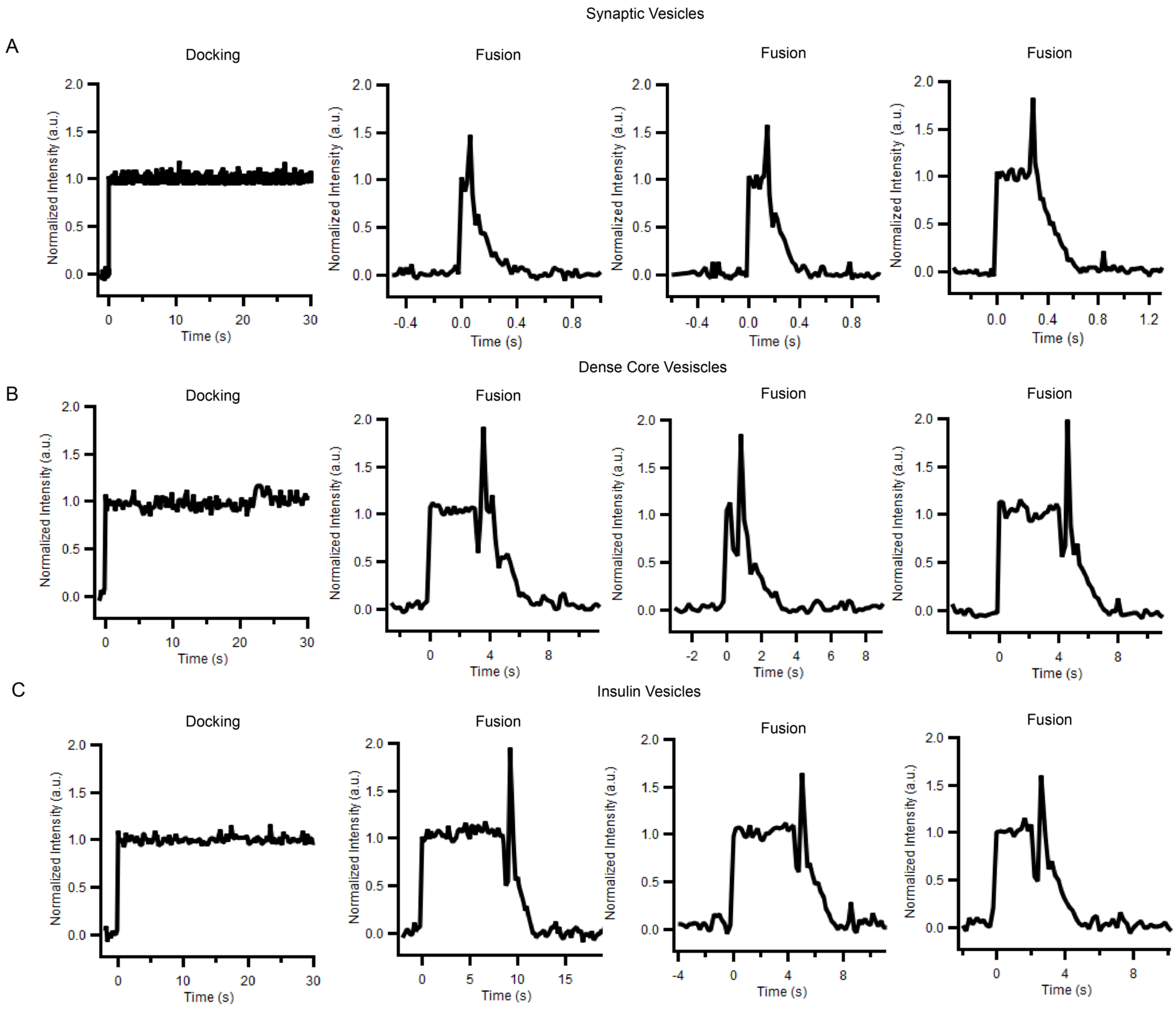

Supplementary Figure 2: Representative docking (left column) and fusion (right three columns) events for acridine orange labeled synaptic vesicles (A), NPY-mRuby labeled dense core vesicles (B), and C-peptide-GFP labeled insulin vesicles (C). The events are similar to those shown in Figure 1 A-C.

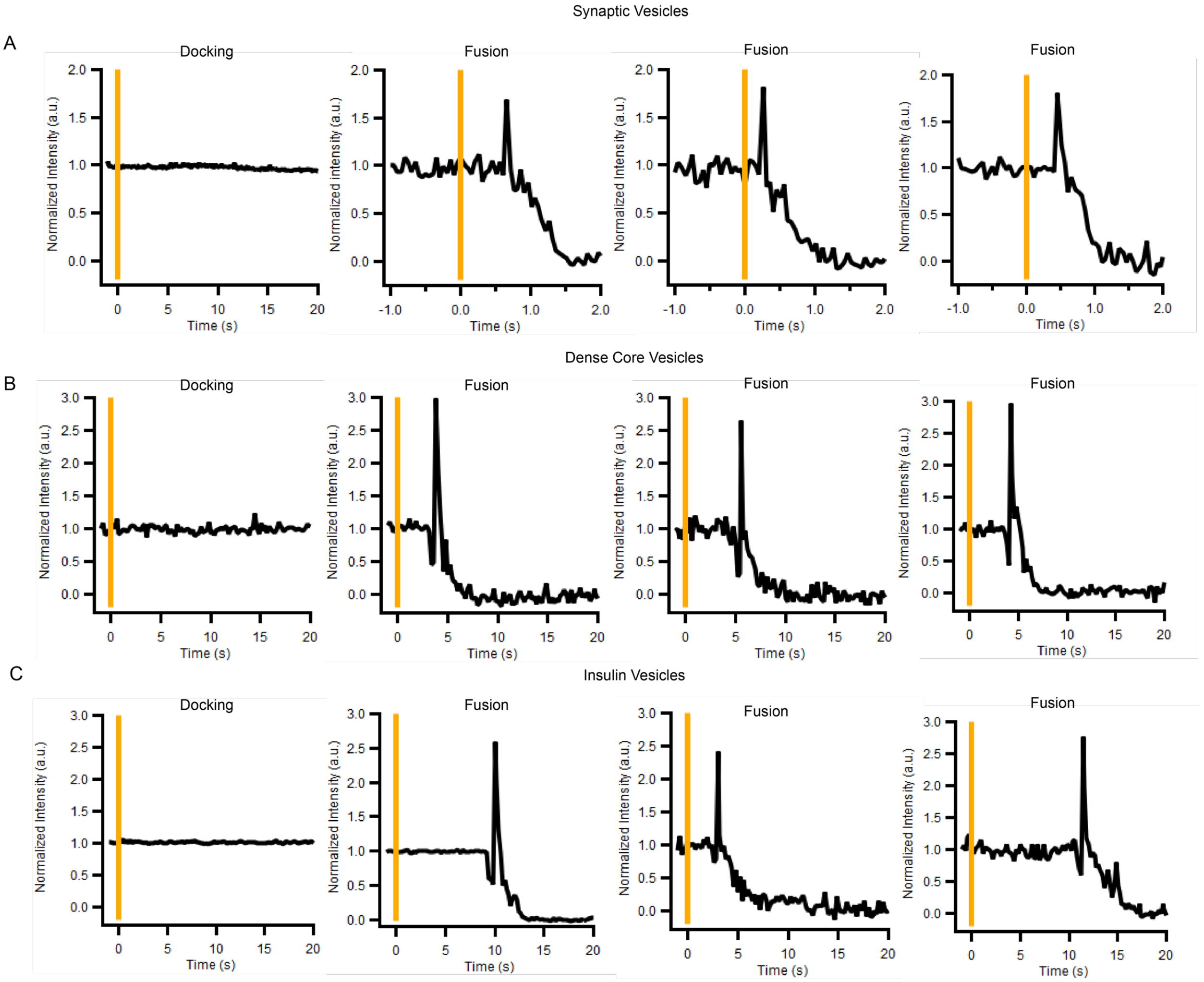

Supplementary Figure 3: Representative traces of single events of calcium triggered fusion of acridine orange labeled synaptic vesicles (A), NPY-mRuby labeled dense core vesicles (B), and C-peptide-GFP labeled insulin vesicles (C). The left column shows vesicle events were docked and did not fuse and the right three columns show events of vesicles that fused in response to calcium. The events are similar to those shown in Figure 3B. The orange line indicated the time calcium was determined to have arrived.

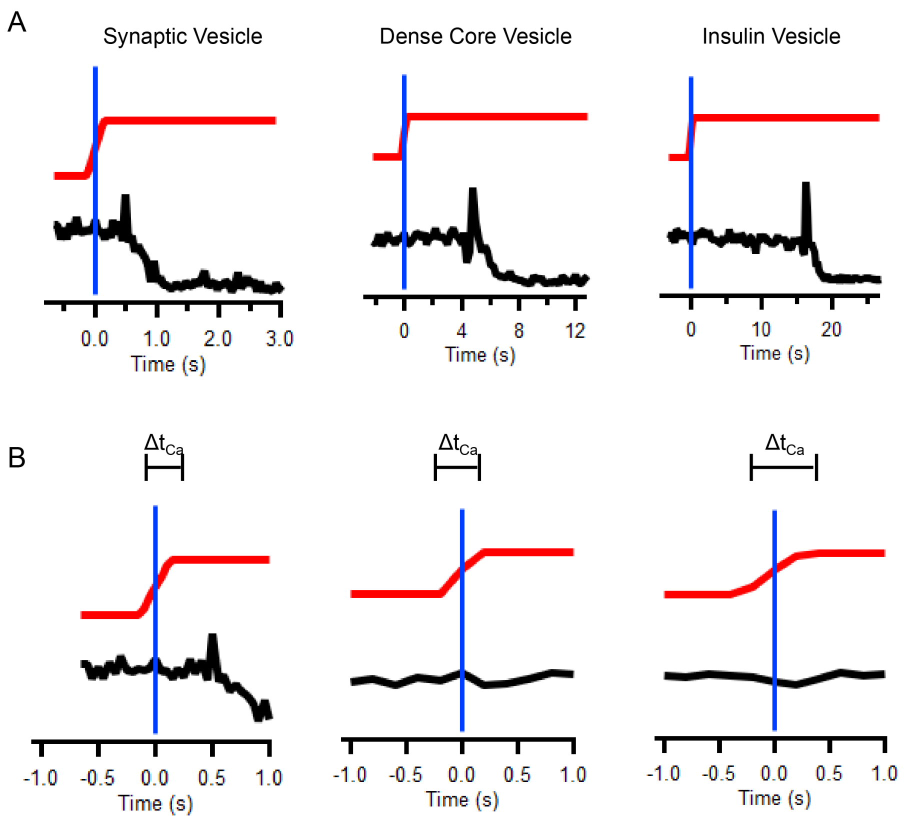

Supplementary Figure 4: Triggered fusion events from Figure 3B presented in the same manner (A) and on an expanded time scale (B) to show the time of buffer exchange with the average buffer exchange times for all experimental conditions shown in Supplementary Table 2. The black lines represent the respective fluorescent content markers and the red lines represent Alexa647 fluorescence marking calcium arrival.

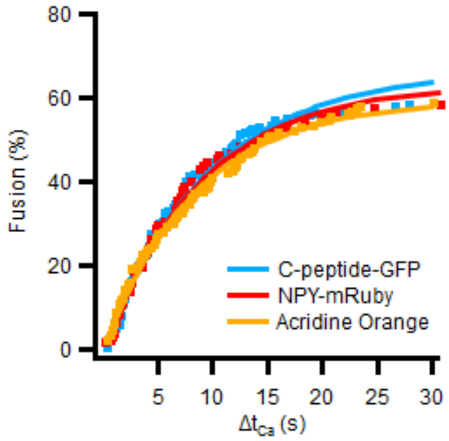

Supplementary Figure 5: Fusion kinetics from time of calcium arrival for insulin vesicles labeled with C-peptide-GFP, NPY-mRuby, or acridine orange. Summary of data is in Supplementary Table 3.

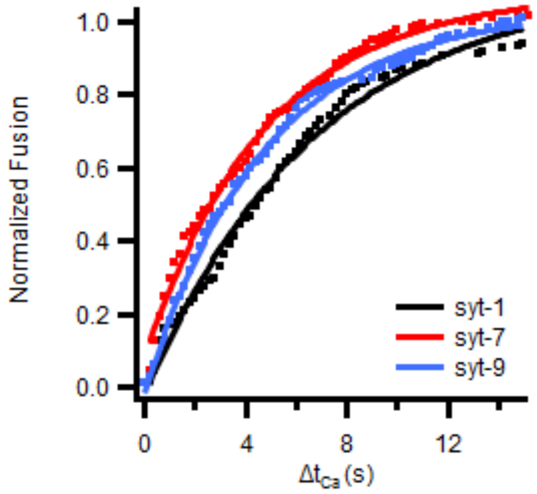

Supplementary Figure 6: Fusion kinetics from time of calcium arrival for dense core vesicles expressing only single synaptotagmin isoforms. Summary of statistics is in Supplementary Table 4.

References:

1. Kreutzberger AJB, Kiessling V, Liang B, Seelheim P, Jakhanwal S, Jahn R, et al. Reconstitution of calcium-mediated exocytosis of dense-core vesicles. Sci Adv. 2017;3(7):e1603208.

2. Takamori S, Holt M, Stenius K, Lemke EA, Gronborg M, Riedel D, et al. Molecular anatomy of a trafficking organelle. Cell. 2006;127(4):831-46.
